# Cognitive flexibility and perceived threat from COVID-19 mediate the relationship between childhood maltreatment and state anxiety

**DOI:** 10.1371/journal.pone.0243881

**Published:** 2020-12-11

**Authors:** Vrinda Kalia, Katherine Knauft, Niki Hayatbini

**Affiliations:** Psychology Department, Miami University, Oxford, Ohio, United States of America; University of Sao Paulo Medical School, BRAZIL

## Abstract

Converging empirical evidence indicates that exposure to adversity in childhood is associated with increased vulnerability to mental health problems in adulthood. As early life adversity has the potential to alter an individual’s appraisal of threat, we hypothesized that individuals exposed to adversity in childhood may also exhibit increased threat from environmental stressors, which in turn may impact their state anxiety levels. We examined the relations between adverse childhood experiences, assessed using the Adverse Childhood Experiences Scale (ACEs), perceived threat from COVID-19, and state anxiety in a sample of adults. Additionally, flexibility is implicated in adaptive coping with life’s stressors so we also assessed participants’ cognitive flexibility. Parallel mediation regression analyses revealed that both perceived threat from COVID-19 and flexibility in the appraisal of challenges mediated the influence of maltreatment, but not household dysfunction, on state anxiety. Our data indicate that experience with early life adversity in the form of maltreatment is associated with increased perceived threat from COVID-19, which results in higher anxiety levels for the individual. In contrast, childhood maltreatment is associated with reduced flexibility in appraising challenges, which in turn mediates the relationship between maltreatment and anxiety. The findings of this study adds to the limited literature on the impact of early life adversity on cognitive flexibility and highlights the psychological toll of COVID-19 on individuals who have been exposed to adverse childhood experiences.

## Introduction

Stressful experiences, such as living through a global pandemic of a highly communicable disease, can have a profound impact on an individual’s life. Initially described as pneumonia with an unidentified cause on 31^st^ December 2019, the outbreak of COVID-19 was declared a Public Health Emergency of International Concern on 20^th^ January, 2020 (Ho et al., 2020). To date, over 44 million individuals worldwide (nearly 9 million in the United States) have tested positive for COVID-19, and over 1.16 million individuals (nearly 227,000 in the United States) have died as result of exposure to the outbreak. Past research with the H1N1 pandemic (also known as ‘swine flu’) has demonstrated that pandemics are associated with increased uncertainty [1] and emotional distress [2]. Some individuals emerge from stressful life events with increased resilience [3, 4] whereas others develop psychopathologies (including depression and anxiety) [5]. This suggests the existence of individual level variability in response to life’s stressors [6]. Considering that stressful life experiences are relatively commonplace, identifying factors that increase vulnerability to life’s stressors or confer resilience in stressful circumstances is essential.

### Early life adversity increases vulnerability

Early life adversity (ELA; hereafter) is implicated in the development of health problems later in life [7], including increased risk of mental illness [5], chronic diseases [8] and reduced life expectancy [9]. Unfortunately, individuals with exposure to ELA are not rare in the American population [10, 11]. According to the Centers for Disease Control and Prevention (CDC), approximately 61% of adults surveyed reported experiencing at least one type of ELA. Additionally, experience with one type of ELA makes it more likely that the individual will report exposure to another, as the different types of adversities (e.g., maltreatment, neglect, household dysfunction) tend to co-occur [5, 7, 9]. For example, if a child grows up in a violent home where they are maltreated they are also more likely to experience neglect from their primary caregivers. As a result, researchers have studied the cumulative effect of adverse experiences along with exposure to different types of adversity [5].

Empirical evidence suggests that ELA alters normative development of the amygdala [12]. Essential for threat detection, the amygdala is engaged more often in a home environment where the child is being maltreated. Over time, this results in sustained enhanced engagement of the amygdala even in environments that are not stressful [6]. For instance, neuroimaging (i.e., fMRI) research has shown that children who have been exposed to family violence exhibit heightened activation in the amygdala when viewing angry faces in comparison to children who have not been maltreated [13]. Although the ability to attend to threats in the environment while attending to ongoing task demands is adaptive [14], exposure to maltreatment biases attentional and emotional processes toward threatening stimuli in the environment [15]. The inability to flexibly attend to environmental demands is associated with a range of psychological disorders, including anxiety [16].

Additionally, ELA can dysregulate the adaptive physiological stress response [17]. Activation of the body’s stress response under acute stress conditions is considered adaptive [18] as it prepares the individual for active engagement with the stressful environmental conditions. However, dysregulation of the stress response results in a disruption of the body’s ability to maintain stability in the face of changing environmental demands, which is known as allostasis [18]. The disruption of allostasis can result in increased vulnerability to stressful events in adulthood [18, 19]. One of the ways in which this vulnerability may be expressed is through heightened sensitivity to perturbations in environmental conditions [20]. For example, a daily diary study with adults who had experienced maternal abuse as children showed that maternal abuse moderated the relationship between stress and affect [21]. Participants with high maternal abuse exposure exhibited a stronger association between daily stressors and negative affect than participants with low maternal abuse exposure. In effect, individuals exposed to ELA are more likely to have a stronger negative reaction to stressors in their environment [22].

### Cognitive flexibility confers resilience

Alongside the study of factors that increase vulnerability to stressful events, psychologists have been interested in identifying factors that enhance an individual’s resilience in the face of stress [23]. Fundamentally, the ability to bounce back from adversity requires adaptation to changing circumstances [24]. Strategies to cope with stressful events must be flexibly applied to changing contextual demands [23, 25]. An example of this is presented in the experimental work by Troy and colleagues [26]. Their work demonstrates that cognitive reappraisal, as a coping strategy, is more effective under conditions of uncontrollable stress than during stressful conditions wherein a person feels they have more control [26]. So, researchers have recognized that being *flexible* in deploying regulatory strategies, that are sensitive to the type of stressor, is more pertinent than discovering the one coping strategy that could be universally successful. Thus, flexibility has emerged as crucial to overall health, wellbeing, and adjustment [23, 27].

Cognitive flexibility is an aspect of flexibility. It is a multifaceted construct, exhibiting both trait and state characteristics [28], and a key component of executive functions [29]. Although difficult to define [30], one definition of cognitive flexibility refers to the ability to switch flexibly between rules or shift between modes of thinking [31]. In order to exhibit this form of cognitive flexibility, an individual must inhibit irrelevant information and deploy attention resources effectively. The ability to switch between set ways of thinking can be particularly useful when an individual confronts a stressor that they cannot control [26]. For instance, cognitive flexibility would allow an individual to reframe their current understanding of a global pandemic (as an uncontrollable stressor) and reconsider behaviors (e.g. hand-washing, wearing a mask) that would help them mitigate their risk in a challenging context [23, 32]. Whereas cognitive flexibility is implicated in problem solving [33, 34] and mechanisms supporting effective regulation of emotions [35]; cognitive *inflexibility* is associated with both maladaptive thought patterns (such as rumination) [36] and anxiety [37]. In laboratory settings, cognitive flexibility is measured using set-shifting tasks such as the Wisconsin Card Sort Test [38] which assess the individual’s ability to switch between rules. Otherwise, self-report measures of cognitive flexibility are an efficient way of measuring cognitive flexibility, particularly state characteristics of cognitive flexibility [39].

### Current study

The brief overview of the literature suggests that exposure to ELA increases sensitivity to environmental stressors. Additionally, individuals exposed to ELA are more likely to appraise environmental stressors as threatening. Finally, cognitive flexibility may allow individuals to respond more adaptively to environmental stressors. Since exposure to ELA enhances the probability of developing anxiety disorders [40] and aberrant threat perception is characteristic to anxiety disorders [41], our primary aim was to examine the relations between ELA, perceived threat from an environmental stressor and anxiety. Because the relation between early adversity and cognitive flexibility is less studied [11], and deficits in cognitive flexibility are associated with anxious behavior [42]; we also investigated the role of cognitive flexibility in the relationship between early life adversity and anxiety.

Since ELA increases vulnerability to the development of anxiety disorders [9, 43, 44], our first prediction was that reported exposure to adverse childhood experiences would be positively correlated with reported state anxiety levels. Additionally, threat appraisal is influenced by ELA [12] so our second prediction was that individuals with experience of early life adversity would perceive COVID-19 as a bigger threat. Reported exposure to adverse childhood experiences would be positively correlated with perceived threat from COVID-19. As prior research has shown that adverse childhood experiences are associated with reduced cognitive flexibility [11], our third prediction was that exposure to adverse experiences would be negatively correlated with cognitive flexibility. Since early maltreatment biases attentional processes toward threatening stimuli [15, 44], our fourth prediction was that perceived threat from COVID-19 would mediate the relationship between early life adversity and anxiety. As prior research has shown that ACEs are associated with reduced cognitive flexibility [11] and lack of flexibility is implicated in higher anxiety [16, 27] our final prediction was cognitive flexibility would mediate the relationship between ACE-maltreatment and ACE-household dysfunction and anxiety.

## Methods

### Participants and procedure

Across 2 days in March (26^th^ and 27^th^) 2020, individuals residing in the United States participated in this study via the online platform Prolific (for details see [45]). At the time of data collection the United States had 160,000 individuals who had tested positive for COVID-19 and nearly 3,000 deaths due attributed to COVID-19. On Prolific participants are able to see advertisements for studies on their homepage. Once they click on a study description, they can opt to participate or not. If they opted to participate they were asked to sign an informed consent form and then taken to our survey. We focused on participants over 18 years of age residing in the United States. Participants were given $4.50 as compensation. The final sample was 356 (3 participants were removed after failing more than 1 attention check; Males = 194, Females = 159, Non-binary = 2, prefer not to describe = 1). Participants were recruited until the maximum number approved by the institutional review board was reached. The target sample size (N = 350) was determined using Monte Carlo simulation anticipating small to medium effect sizes. Participants’ age ranged from 18–80 years (*M*_*Age*_ = 36.50, 95% were younger than 61 years). A majority of the participants identified as White (80.1%) and the remaining participants identified as Asian (5.1%), Hispanic (5.9%), African American (6.5%), and Native American (0.3%), Indian American (0.6%), a race or ethnicity not listed (0.6%), or preferred not to disclose (0.3%). All study procedures were approved by the Institutional Review Board. Following informed consent, participants completed the questionnaires in this order: cognitive flexibility inventory (CFI), state trait anxiety inventory (STAI short form), adverse childhood experiences scale (ACEs), and perceived threat of COVID-19, assessed through a series of questions embedded in the demographics section of the survey.

### Measures

#### Adverse childhood experiences scale (ACEs) [46]

Although the exact definition of child adversity is debated [47], one way to capture an individual’s exposure to early life adversity is through the Adverse Childhood Experiences scale (ACEs) [46]. Developed by researchers from Kaiser Permanente in collaboration with researchers at Centers for Disease Control and Prevention (CDC), the ACE scale measures an individual’s exposure to adverse experiences in early development (i.e., prior to 18 years of age). Recognition of the co-occurrence of types of ELA has received much deserved attention in the literature through the cumulative stress model [48, 49]. However, we were interested in the mechanisms underlying the development of threat and fear processes associated with anxiety, so we considered whether the effect of ELA would be different based on the dimensions of threat and deprivation that have previously been proposed [50]. For the purposes of this study ELA, as measured by the ACEs, was split into two types of adversity—maltreatment (which includes physical and emotional neglect) and household dysfunction. Recent research using confirmatory factor analysis has shown that these two types of adversity can be identified as the two factors underlying the ACEs [51].

The ACE scale [46] we used is a 10-item questionnaire that assesses exposure to early life adversity prior to 18 years of age. Five of the 10 questions ask about experiences with maltreatment (emotional, physical, and sexual abuse and neglect; (e.g., “*Did a parent or other adult in the household often swear at you*, *insult you*, *put you down*, *or humiliate you*? *Or*, *act in a way that made you afraid that you might be physically hurt*?”), and the other 5 ask about household dysfunction (domestic violence, parental separation or divorce, and the presence of a substance-abusing, mentally ill; e.g., “*Was a household member depressed or mentally ill*, *or did a household member attempt suicide*?”). Each question was presented in a dichotomous scale (yes/no). Every response in the affirmative (i.e. yes) to a question was given 1 point, and these were summed to calculate scores of an individual’s exposures to maltreatment and household dysfunction. Higher scores indicate greater exposure to maltreatment and household dysfunction. Cronbach alpha for maltreatment (α = .78) and household dysfunction (α = .69) were acceptable.

#### State-trait anxiety inventory (STAI) [52]

Our primary outcome variable was state anxiety, which were assessed using the state form of the State Trait Anxiety Inventory [52]. The original inventory is a 20-item self-report instrument developed to assess levels of situation-related (state) anxiety. To reduce the burden on our participants, we used the short form of STAI [52] which has 6 items. Prior reports have shown that scores of the 6-item and full-length inventory are strongly correlated, *r* = .95 [53]. One example of this inventory is “*I am worried*”. Items are rated on a 4-point Likert-type scale (*1 = “Not at all”*, *2 = “Somewhat”*, *3 = “Moderately so”*, *4 = “Very much so”*) to produce a summative score ranging from 6 to 24 with higher scores indicating higher levels of anxiety (α = .90).

#### Cognitive flexibility inventory (CFI) [54]

Since maladaptive cognitions are characteristic of anxiety disorders [55] we were particularly interested in examining the type of cognitive flexibility that would facilitate the removal and replacement of these maladaptive thought patterns. So, we assessed cognitive flexibility using the self-report measure Cognitive Flexibility Inventory (CFI) [54]. The CFI measures two aspects of cognitive flexibility—alternatives (i.e., coming up with alternative solutions to problems) and control (i.e., experiencing challenges as within one’s control). The CFI [54] is a 20-item scale that measures an individual’s ability to successfully challenge and replace maladaptive thoughts with more balanced and adaptive thinking when encountering challenges or stressors in life. Each response is made on a seven-point Likert scale ranging from *strongly disagree* (1) to *strongly agree* (7). As described in the original manuscript that detailed the development of the CFI, we reverse scored appropriate items and then summed up the responses to the likert scale items obtain a total score [54]. Higher scores on the two aspects of CFI indicate greater cognitive flexibility. The inventory measures two types of cognitive flexibility: 1) Alternatives which is defined as the ability to perceive multiple alternative explanations for life occurrences, and the ability to generate multiple alternative solutions to difficult situations (e.g., *I consider multiple options before responding to difficult situations;* α = .90), and 2) Control which is defined as the tendency to perceive difficult situations as controllable (e.g., *When I encounter difficult situations*, *I feel like I am losing control;* α = .80).

#### Perceived threat of COVID-19

Finally, as threat perception is related to anxiety [56] and influenced by early life adversity [12], we sought to study the relationship between early life adversity, perceived threat from an environmental stressor, and anxiety. Based on past research with the H1N1 pandemic [2] and the fact that our data were collected in the midst of the COVID-19 pandemic, we operationalized the perception of threat from environmental stressors as an individual’s perception of the threat posed by the COVID-19 virus.

Participants responded to the questions about their perception of the severity of the problem of the COVID-19 outbreak through two questions that were adapted from previous research on the perception of pandemics [57]: 1) How much have you been impacted by COVID-19? (1 = a little to 5 = my life has completely changed) and 2) How serious of a problem do you think COVID-19 is? (1 = it’s not very serious to 5 = it is catastrophic). Additionally, based on prior published research on the H1N1 pandemic [1] participants were asked about their perceived susceptibility through one question: 1) How likely is it that you would test positive for COVID-19? (1 = not very likely to 5 = extremely likely). All questions were posed using a 5-point Likert scale. Ratings on the three items were summed up as an indicator of their perceived threat from the outbreak.

### Data analytic plan

In order to interrogate the relationship between ACEs, perceived threat from COVID-19, cognitive flexibility and state anxiety levels, we considered mediation models with ACEs-maltreatment and ACEs-family dysfunction as predictor variables and state anxiety as the outcome variable. As we expected childhood experiences of maltreatment and household dysfunction to have differential effects on anxiety [50], we ran two models. In both mediation models, CFI-Control and perceived threat from COVID-19 were added simultaneously as parallel mediators. Thus, for each of these models, either maltreatment or household dysfunction was the independent variable, threat perception and CFI-Control acted as parallel mediators, and state anxiety was the dependent variable. In prior research gender [58] has emerged as a significant covariate predicting state anxiety, with women reporting higher levels of state anxiety than men so we controlled for gender in our models. Additionally, we controlled for age as there is evidence that older individuals exhibit more distress during pandemics [2]. Finally, education and socioeconomic status are associated with anxiety [59], so we controlled these variables as well in the analyses.

**Fig 1:**
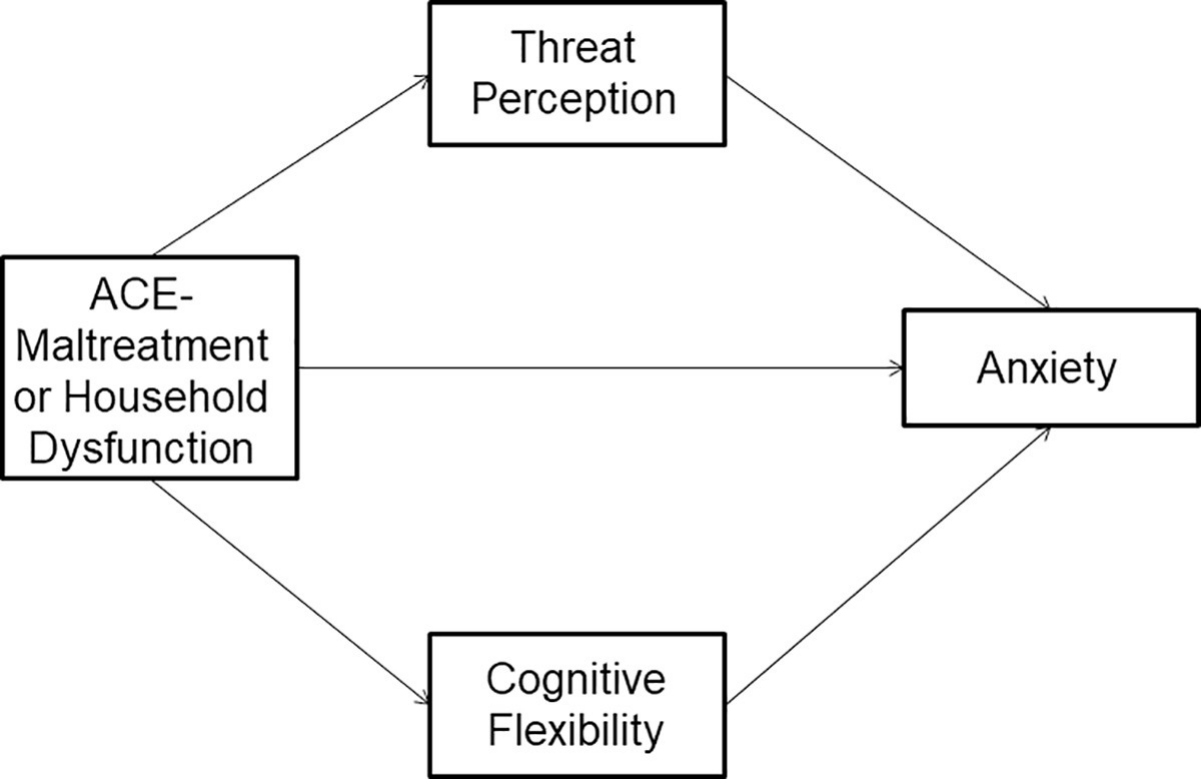
Proposed parallel mediation model. Conceptual parallel mediation model in which the independent variable (ACE-maltreatment or household dysfunction) impacts the dependent variable (Anxiety) through the two parallel mediators (threat perception and CFI control).

We used Hayes’ PROCESS 3 macro model 4 [60] in SPSS version 25.0 to conduct these analyses. This method uses boot-strapping and ordinary least-squares regression-based analyses to simultaneously test the parallel indirect effects within the model. This analytic strategy would ultimately help identify the degree to which cognitive flexibility and threat perception simultaneously mediate the relationship between adverse childhood experiences and current anxiety levels. From a process viewpoint, parallel mediators are influenced by the predictor variable (i.e., ACEs-maltreatment or ACEs-household dysfunction) and impact the outcome variable (i.e., anxiety) but do not influence each other (i.e., cognitive flexibility and perceived threat from COVID-19); although the two mediators are allowed to be correlated with one another. See Fig 1.

## Results

### Descriptive statistics and correlations

All variables were normally distributed (skew coefficient < |2|; kurtosis coefficient < |3|). Bivariate correlations and descriptive statistics for the variables of interest are presented in Table 1. Some participants (n = 7) did not respond to threat perception questions and one participant did not respond to questions on the ACE-maltreatment subscale, so their results are not included in the relevant models.

**Table 1:** Prevalence of each category of adverse childhood experiences and ACE score by gender.

		Number (%)		
		Women (n = 158)	Men (n = 194)	Total (N = 356)
Adverse childhood experiences				
	Emotional abuse	73 (45.9)	64 (33.0)	139 (39.0)
	Physical abuse	39 (24.5)	50 (25.8)	90 (25.3)
	Sexual abuse	23 (14.5)	13 (6.7)	36 (10.1)
	Physical neglect	21 (13.2)	24 (12.4)	46 (12.9)
	Emotional neglect	48 (30.2)	38 (19.6)	88 (24.7)
	Parental separation or divorce	62 (39.0)	62 (32.0)	126 (35.4)
	Battered parent	33 (20.8)	22 (11.3)	56 (15.7)
	Household alcohol/drug abuse	50 (31.4)	29 (14.9)	80 (22.5)
	Mental illness in household	52 (32.7)	34 (17.5)	88 (24.7)
	Incarcerated family member	10 (6.3)	20 (10.3)	30 (8.4)
Adverse childhood experiences score				
	0	48 (30.2)	78 (40.2)	126 (35.4)
	1	26 (16.4)	34 (17.5)	61 (17.1)
	2	20 (12.6)	31 (16.0)	51 (14.3)
	≥ 3	65 (41.1)	51 (26.3)	118 (33.1)

Note. Three participants identified their gender as non-binary or preferred to self-describe.

**Fig 2:**
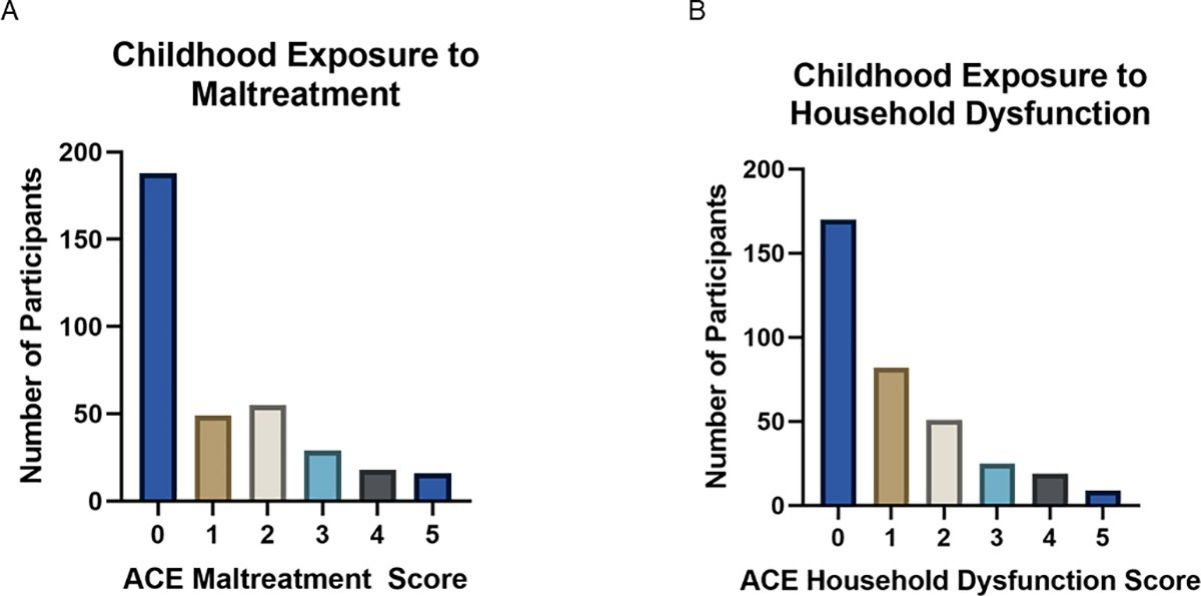
Frequencies of reported ACE scores. Observed frequencies of ACE-Maltreatment (A) and ACE-Family Dysfunction scores within the sample. Each score represents the total number of types of maltreatment or family dysfunction reported by the individual, rather than cumulative occurrences.

Consistent with prior studies our data indicate that approximately 47% of the adults in our study reported experiencing at least one ACE-maltreatment with 52% reporting exposure to at least one ACE-household dysfunction. See Fig 2 and Table 1.

### Regression models

To assess effects of ACEs as a whole and simultaneous effects of both maltreatment and household dysfunction on the proposed mediators and dependent variable, we ran regression models. Four models included covariates and ACE total scores, and four included covariates, maltreatment, and household dysfunction. Consistent with correlational evidence (See Table 2), neither model with Alternatives as the dependent variable accounted for significant variance in CFI-Alternatives (*p* > .09) The remaining models including ACE total scores suggest that ACEs are significantly associated with the proposed mediators and dependent variable. Greater numbers of total ACEs were associated with greater threat perception, *B* = 0.11, *t*(343) = 2.32, *p* = .021, and anxiety, *B* = 0.25, *t*(350) = 2.58, *p* = .010, over and above the effects of covariates. In contrast, greater total ACE scores were associated with reduced CFI Control, *B* = -0.43, *t*(350) = -2.73, *p* = .007. When both maltreatment and household dysfunction were instead included in a model, neither significantly predicted CFI-Control nor Anxiety over and above the other (*p*s > .09). This may be due to the moderate levels of shared variance between maltreatment and household dysfunction (See Table 2). Despite this high level of shared variance, maltreatment significantly predicted greater threat perception, *B* = 0.31, *t*(341) = 2.42, *p* = .016, while household dysfunction was not associated with threat perception when maltreatment is included in the model. Together, this suggests that, while there are many ways that maltreatment and household dysfunction may have similar effects, the two factors differ in their relationship with threat perception. See Table 3.

**Table 2:** Bivariate correlations and descriptive statistics.

	1.	2.	3.	4.	5.	6.	7.	8.	9.
1. Maltreatment	-								
2. Household Dysfunction	.62***	-							
3. Threat Perception	.12*	.08	-						
4. CFI-Control	-.13*	-.13*	-.17**	-					
5. CFI-Alternatives	.05	.05	.11*	.24***	-				
6. Anxiety	.14*	.14*	.33***	-.47***	-.16**	-			
7. Age	.06	.05	.00	.29***	.03	-.11*	-		
8. Education	-.15**	-.14**	.16**	.08	.08	.00	.16**	-	
9. SES	.10*	-.08	.01	.08	.14**	-.07	-.02	.43***	-
*Mean*	1.12	1.07	8.79	31.32	74.23	12.89	36.50	4.45	5.46
*SD*	1.48	1.34	2.11	7.64	9.50	4.58	12.41	1.47	1.69
*Range*	0–5	0–5	4–14	10–44	46–91	6–24	18–80	1–8	1–10

* *p* < .05

** *p* < .01

*** *p* < .001. Education: 1 = Some high school, 8 = Doctorate (PhD, MD, etc.).

**Table 3:** Regression models of relationships between ACEs total scores, maltreatment, and household dysfunction with proposed mediators and the outcome variable.

**Total ACEs Models**	**Outcome Variables**							
	*Threat Perception*		*CFI Control*		*CFI Alternatives*		*Anxiety*	
	R^2^ = .06**		R^2^ = .12***		R^2^ = .03		R^2^ = .07***	
	B	SE	B	SE	B	SE	B	SE
Gender	0.40	0.21	-1.25	0.74	0.28	0.89	1.35**	0.46
Age	-0.01	0.01	0.19***	0.03	0.03	0.04	-0.05*	0.02
Education	0.31***	0.07	-0.12	0.30	-0.18	0.36	0.26	0.19
SES	-0.09	0.07	0.35	0.25	0.79**	0.30	-0.25	0.16
ACEs Total Score	0.11*	0.05	-0.43**	0.16	0.27	0.19	0.25*	0.10
**Maltreatment & Household Dysfunction Models**	**Outcome Variables**							
	*Threat Perception*		*CFI Control*		*CFI Alternatives*		*Anxiety*	
	R^2^ = .07***		R^2^ = .12***		R^2^ = .03		R^2^ = .07***	
	B	SE	B	SE	B	SE	B	SE
Gender	0.43*	0.21	-1.12	-.74	0.47	0.88	1.36**	0.46
Age	-0.01	0.01	0.19***	0.03	0.03	0.04	-0.05*	0.02
Education	0.31***	0.09	-0.16	0.30	-0.25	0.36	0.24	0.18
SES	-0.08	0.07	0.32	0.25	0.75	0.30	-0.26	0.16
Maltreatment	0.31*	0.13	-0.28	0.43	0.30	0.39	0.14	0.27
Household Dysfunction	-0.04	0.10	-0.56	0.34	0.17	0.44	0.32	0.21

Note.

* p < .05

** p < .01

*** p < .001; B is the unstandardized coefficient; These data come from eight regressions in which ACEs scores or the two factors were entered simultaneously along with covariates as predictors of each outcome.

### Parallel mediation model: Maltreatment as predictor

#### Maltreatment as a predictor of mediators

Maltreatment and the covariates accounted for 6% of variance in threat perception (See Table 4), *R*^2^ = .06, *F*(5, 342) = 4.52, *p* < .001. Maltreatment, *B* = 0.19, *t*(342) = 2.47, *p* = .014, and education, *B* = 0.31, *t*(342) = 3.60, *p* < .001, emerged as significant predictors of threat perception. Exposure to maltreatment and higher education levels predicted elevated threat perception. Maltreatment and the covariates accounted for 12% of the variance in CFI-Control, *R*^2^ = .12, *F*(5, 342) = 8.92, *p* < .001. Maltreatment, *B* = -.71, *t*(342) = -2.63, *p* = .009, and age, *B* = 0.19, *t*(342) = 5.84, *p* < .001, emerged as significant predictors of CFI-Control. Maltreatment predicted lower levels of CFI-Control whereas older individuals had higher levels of CFI-Control.

**Table 4:** Regression models of the relationship between ACE-maltreatment and mediators.

Variable entered	Threat Perception		CFI Control	
	R^2^ = .06**		R^2^ = .12***	
	B	SE	B	SE
ACEs Maltreatment	0.19*	0.54	-0.71**	0.27
Age	-0.01	0.01	0.19***	0.03
Education	0.31***	0.09	-0.13	0.30
SES	-0.08	0.07	0.30	0.25
Gender	0.41	0.21	-1.12	0.74

Note.

* p < .05

** p < .01

*** p < .001; B is the unstandardized coefficient.

#### Parallel mediation model

**Fig 3:**
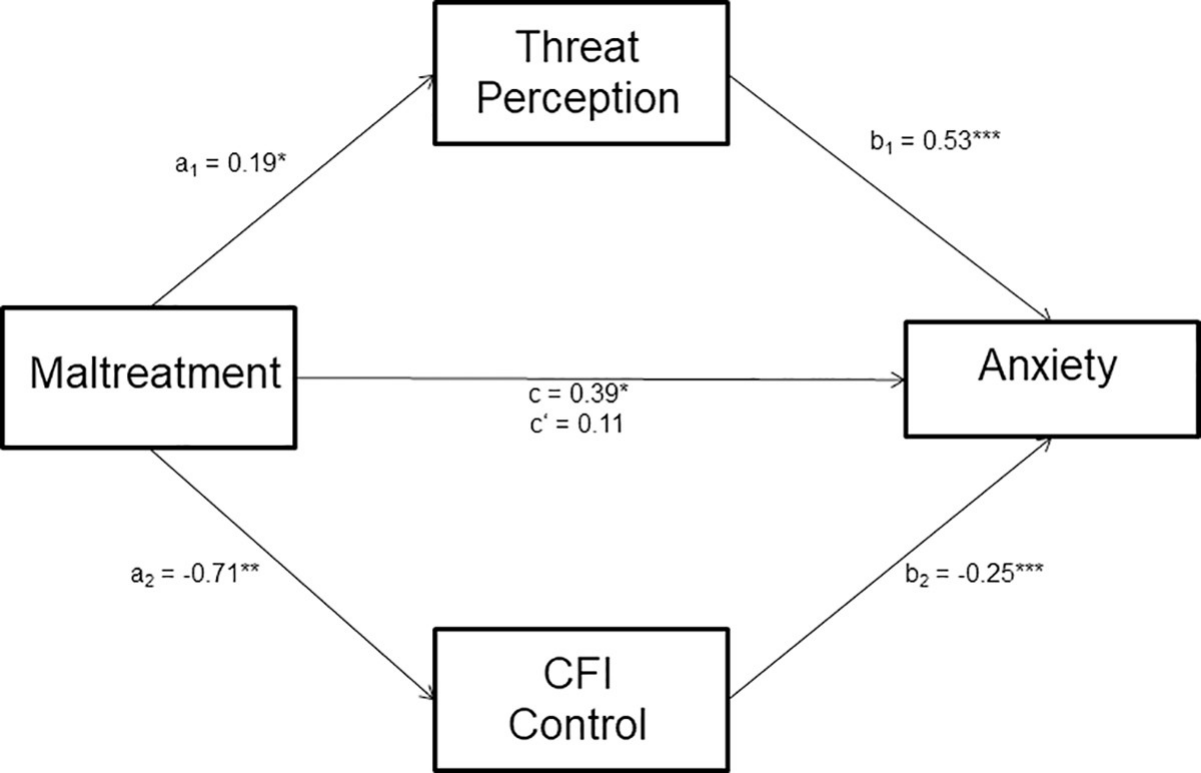
ACE-maltreatment parallel mediation model. Mediation analyses from parallel mediation of maltreatment on anxiety through threat perception and CFI control. Path a_1_ is the effect of maltreatment on the proposed mediator threat perception, path a_2_ is the effect of maltreatment on the proposed mediator CFI-Control. Path b_1_ shows the effect of threat perception on anxiety, while path b_2_ shows the effect of CFI-Control on anxiety. Path c shows the total effect of maltreatment on anxiety when the mediators are not included in the model. In contrast, path c’ shows the direct effect of maltreatment on anxiety over and above the two mediators. *p < .05; **p < .01; ***p < .001.

Maltreatment, the mediators (threat perception and CFI-Control), and the covariates accounted for 30% of the variance in state anxiety, *R*^2^ = .30, *F*(7, 340) = 20.96, *p* < .001. See Fig 3. Threat perception, *B* = 0.53, *t*(340) = 5.15, *p* < .001, and CFI-Control, *B* = -0.25, *t*(340) = -8.59, *p* < .001, emerged as significant predictors of anxiety. The total effect of maltreatment on anxiety was significant, *B* = 0.39, *t*(342) = 2.35, *p* = .020. However, when mediators were included in the model, the direct effect of maltreatment on anxiety was no longer significant, *B* = 0.11, *t*(340) = 0.77, *p* = .443. The effect of maltreatment on state anxiety was fully mediated by threat perception and cognitive flexibility. A 95% bias-corrected confidence interval based on 10,000 bootstrap samples indicated that the indirect effect through threat perception, *B* = 0.10, *SE* = 0.05, 95% CI: [.014, .197], was entirely above zero. Similarly, the indirect effect through CFI-Control, *B* = 0.18, *SE* = 0.09, 95% CI: [.003, .343] was also above zero. Gender emerged as a significant predictor of state anxiety, *B* = 0.88, *t*(340) = 2.20, *p* = .029, such that women reported higher levels of anxiety than men.

### Parallel mediation model: Household dysfunction as predictor

#### Household dysfunction as a predictor of mediators

Household dysfunction and the covariates accounted for 5% of variance in threat perception (See Table 5), *R*^2^ = .05, *F*(5, 343) = 3.81, *p* = .002. However, only education, *B* = 0.29, *t*(343) = 3.43, *p* < .001, and gender, *B* = 0.42, *t*(343) = 1.98, *p* = .049, predicted elevated threat perception. Both women and individuals with more education tended to report higher threat perception. Unlike maltreatment, household dysfunction did not influence threat perception. Household dysfunction and the covariates accounted for 11% of the variance in CFI-Control, *R*^2^ = .11, *F*(5, 343) = 8.75, *p* < .001. Household dysfunction, *B* = -0.69, *t*(343) = -2.31, *p* = .022, and age, *B* = 0.19, *t*(343) = 5.78, *p* < .001, predicted CFI-Control. Greater household dysfunction was related to lower levels of CFI-Control, and older individuals reported greater CFI-Control.

**Table 5:** Regression models of the relationship between ACE-maltreatment and mediators.

Variable entered	Threat Perception		CFI Control	
	R^2^ = .05**		R^2^ = .12***	
	B	SE	B	SE
Household dysfunction	0.14	0.09	-0.69**	0.30
Age	-0.01	0.01	0.19***	0.03
Education	.29***	0.09	-0.07	0.30
SES	-0.08	0.07	0.33	0.26
Gender	0.42*	0.21	-1.13	0.75

Note.

* p < .05

** p < .01

*** p < .001; B is the unstandardized coefficient.

#### Parallel mediation model

Household dysfunction, the mediators (threat perception and CFI-Control), and the covariates accounted for 29% of the variance in state anxiety, *R*^2^ = .29, *F*(7, 341) = 19.97, *p* < .001. Threat perception, *B* = 0.53, *t*(341) = 5.11, *p* < .001, and CFI-Control, *B* = -0.25, *t*(341) = -8.34, *p* < .001, emerged as significant predictors of anxiety. The total effect of maltreatment on anxiety was significant, *B* = 0.38, *t*(343) = 2.03, *p* = .043. When both mediators were included in the model, the direct effect of household dysfunction on anxiety was no longer significant, *B* = 0.13, *t*(341) = 0.81, *p* = .416. However, the effect of household dysfunction on anxiety was not mediated by threat perception or cognitive flexibility. A 95% bias-corrected confidence interval indicated that the indirect effect of threat perception, *B* = 0.07, *SE* = 0.06, 95% CI: [-.035, .191] contained zero. Similarly, the indirect effect through CFI-Control, *B* = 0.17, *SE* = 0.09, 95% CI: [-.025, .350] also contained zero. Within this model, gender emerged as a predictor of anxiety, *B* = 0.82, *t*(341) = 2.01, *p* = .045, such that women reported higher levels of anxiety than men.

## Discussion

**Fig 4:**
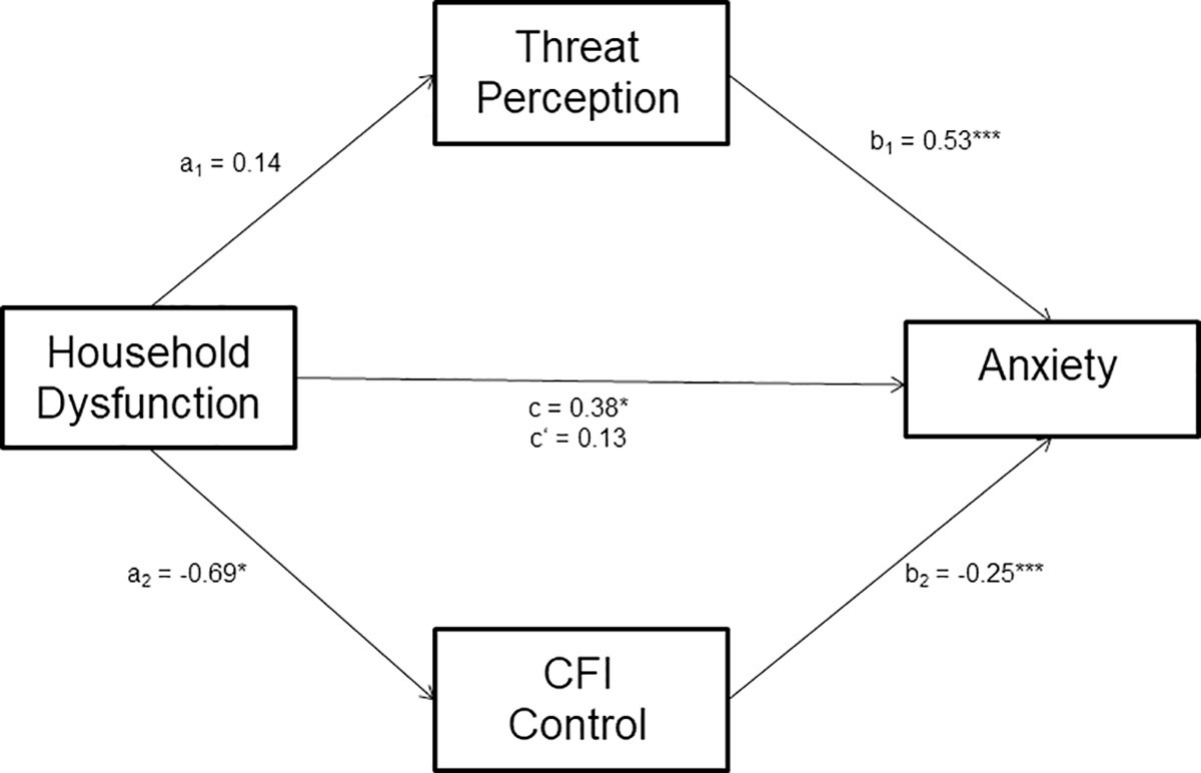
ACE-household dysfunction parallel mediation model. Parallel mediation of ACE-household dysfunction on anxiety through threat perception and CFI control. Path a_1_ is the effect of family dysfunction on the proposed mediator threat perception, path a_2_ is the effect of family dysfunction on the proposed mediator CFI-Control. Path b_1_ shows the effect of threat perception on anxiety, while path b_2_ shows the effect of CFI-Control on anxiety. Path c shows the total effect of ACE-household dysfunction on anxiety when the mediators are not included in the model. In contrast, path c’ shows the direct effect of ACE-household dysfunction on anxiety over and above the two mediators. *p < .05; **p < .01; ***p < .001.

The mediation analyses revealed a significant parallel mediation model, predicting reported anxiety levels, with ACE-maltreatment as the predictor variable and CFI-Control and perceived threat from COVID-19 as parallel mediators. See Fig 3. However, the mediation model with household dysfunction as the predictor and perceived threat from COVID-19 and CFI-Control as mediators (see Fig 4) was not supported by the data.

By studying perceived threat from COVID-19 we were able to show that those individuals who had been exposed to maltreatment, early in development, were also more likely to perceive a greater threat from COVID-19. Further, this perceived threat from COVID-19 fully mediated the relationship between maltreatment and state anxiety. Individuals with exposure to maltreatment had high levels of anxiety which was influenced by their perception of the threat that COVID-19 posed, as well as, their ability to flexibly appraise challenges. To the best of our knowledge we are the first to report these relationships between ACEs, perceived threat, cognitive flexibility and anxiety. It is important to note that, though the study was adequately powered for the analyses we presented, replication of this novel finding with a larger sample is needed before any firm conclusions are drawn.

Our results demonstrate that individuals exposed to maltreatment reported greater perceived threat from COVID-19. This finding suggests that those exposed to a threatening environment in early development were more likely to perceive COVID-19 as an environmental threat. This is consistent with prior research showing that adults who have been maltreated as children exhibit enhanced sensitivity to emotionally salient [61] and threatening stimuli in the environment [62]. Because face processing is foundational to social interaction [63] most of the previous research has used emotion faces in behavioral paradigms to examine threat perception. By studying perceived threat from COVID-19 we were able to extend the current literature on this topic. Separating the ACEs into maltreatment and family dysfunction allowed us to observe that, unlike maltreatment, household dysfunction was unrelated to perceived threat from COVID-19. Our study provides additional support for the notion that experiences of threat and deprivation exhibit differential associations with developmental outcomes [64]. Overall, our data bolsters the notion that exposure to early abuse alters threat appraisal in adulthood [9].

Our data show that experience with early life adversity in the form of maltreatment and household dysfunction was positively correlated with higher anxiety levels during a global pandemic. As such, individuals who reported one or more ACEs for maltreatment and household dysfunction were also more likely to report higher anxiety levels. These data are consistent with prior reports demonstrating that early life adversity, as measured by ACEs, is associated with psychological distress in adulthood [11, 65, 66]. Our study provides additional support for a dosage effect, such that greater exposure to different types of early adversity increases psychological distress [67]. Since the ACEs is a retrospective measure, it is prone to error due to its reliance on the individual’s memory [68], therefore we recommend that longitudinal prospective studies are conducted to validate the reported associations in this paper. In particular, the data we presented are correlational, so no causal claims can be made from this study.

Our study also revealed that exposure to maltreatment enhanced perceived threat from COVID-19 which, in turn, predicted increased levels of anxiety. Anxiety in stressful circumstances, such as a global pandemic, can be protective as it focuses attention toward threatening stimuli in the environment [41]. However, past research has shown that enhanced vigilance to threatening environmental stimuli plays a key role in the development of anxiety disorders [69]. Considering that participants exposed to maltreatment also expressed enhanced threat from COVID-19, our data suggest that these individuals may be vulnerable to developing anxiety disorders. Although we were able to show that state anxiety levels were influenced by the threat perceived from the COVID-19 pandemic, it is important to note that we did not measure trait anxiety levels in our sample. Therefore, it will be important to replicate these findings with a study that takes into account trait level individual differences in anxiety before our result can be extended to clinical populations.

In addition to perceived threat from COVID-19, our data demonstrated that exposure to maltreatment reduced the individual’s ability to flexibly appraise challenges, an aspect of cognitive flexibility measured by CFI-Control. This relation was also observed between household dysfunction and CFI-Control and presents a contrast to the observed relation between ACEs and perceived threat. Our work replicates prior research showing that ACEs predict lower levels of cognitive flexibility as measured by the CFI-Control [11] and provides support for the notion that exposure to ELA alters normative development of executive processes [17]. Since CFI-Control is an indicator of the individual’s ability to flexibly view stressors as challenges (i.e. something within one’s control), our data suggests that ELA makes it more likely that an individual will view challenging circumstances in their daily life as threatening. This interpretation of daily difficulties could make them more reactive to everyday stressors and may be an indicator of increased allostatic load [18]. Thus, it is possible to speculate that exposure to ELA may have developed brain regions associated with vigilance to threats in the environment [9] at the cost of brain regions that are implicated in flexibile thinking.

Contrary to our prediction, we did not observe an association between ACEs and both facets of cognitive flexibility assessed by the CFI. Specifically, neither maltreatment or household dysfunction were associated with CFI-Alternatives. This is consistent with a prior report showing that reported ACEs were unrelated to CFI-Alternatives [11] and provides further proof for the notion that ELA may not influence all aspects of cognitive flexibility in adults. It is worth noting that we only used self-report measures in our study, so it is possible that our findings may not replicate if behavioral measures of cognitive flexibility were used. Regardless, our study adds to the literature on the impact of early life adversity on cognitive flexibility.

Further, cognitive flexibility fully mediated the relationship between maltreatment and anxiety. Specifically, reduction in flexible appraisal, associated with early maltreatment, had an negative impact on the individual’s state anxiety. It is important to note that low scores on CFI-Control indicate inflexibly perceiving all challenging situations as uncontrollable. According to some researchers, viewing future events as controllable can indicate a belief that the individual has the resources needed to cope with the potentially aversive event whereas uncontrollability implies that the event will remain unchanged regardless of the individual’s actions [41]. Our data suggest that individuals who have experienced maltreatment are more likely to view future aversive situations as uncontrollable and this increases their anxiety levels. It is relevant to point out that if an individual does not believe that their problems have any resolution, they will be more likely to engage in maladaptive coping, such as avoidance or rumination [54]. Although these coping strategies may alleviate distress in the short-term, long-term they are more likely to exacerbate any psychological distress the individual is experiencing [70]. Thus, our work provides further evidence that abuse as ELA increases the likelihood of developing an anxiety disorder [71].

### Implications and future directions

Adverse childhood experiences refer to potentially traumatic experiences that unfortunately too many American children have to endure. Overall, our data indicate that maltreatment, as a type of adverse childhood experience, predicts greater sensitivity to threat in the environment and this results in increased anxiety levels during a global pandemic. Brain imaging research has demonstrated that the hypervigilance to threatening stimuli, in maltreated individuals, may be the consequence of increased activity in the amygdala [13, 62]. We did not conduct brain imaging work for our study, but our data should provide impetus for researchers to examine amygdala activity in response to COVID-19 particularly in individuals with early life adversity in the form of maltreatment.

Additionally, our data suggest that reduced cognitive flexibility in individuals who have been exposed to maltreatment may make them vulnerable to developing anxiety disorders following a stressful experience. Thus, enhancing cognitive flexibility in adults who have been maltreated as children might be a promising avenue for treatment programs. Future research should systematically examine the impact of early maltreatment on cognitive flexibility with implications for psychological distress. Most importantly, our data suggest that individuals who have experienced early life adversity are vulnerable to the impact of COVID-19 and need to be considered when examining the psychological toll of the pandemic on our communities.
